# Effect of Polyether Ether Ketone Melt Fluidity on Crystallization Behavior of Carbon Fiber Reinforced Polyether Ether Ketone Composites

**DOI:** 10.3390/molecules31111810

**Published:** 2026-05-25

**Authors:** Weifeng Liu, Xiaran Miao, Shiwen Tao, Ji Li, Jianzhong Ma, Jinjun Yang, Hui Li

**Affiliations:** 1 State Key Laboratory for Modification of Chemical Fibers and Polymer Materials, Center for Advanced Low-Dimension Materials, College of Materials Science and Engineering, Donghua University, Shanghai 201620, China; l1792814476@163.com (W.L.); 17856031513@163.com (S.T.); li17261489882@163.com (J.L.); majzh@avic.com (J.M.); 2 Shanghai Synchrotron Radiation Facility, Shanghai Advanced Research Institute, Chinese Academy of Sciences, Shanghai 201204, China; 3 Avic Composite Co., Ltd., Beijing 101300, China

**Keywords:** CF/PEEK composites, melt flow rate, cooling rate, non-isothermal crystallization kinetics, crystal morphology

## Abstract

The non-isothermal crystallization behavior of CF/PEEK composites during the cooling stage of processing significantly influences their final properties. However, the effect of PEEK melt fluidity on the crystallization kinetics and crystal morphology of CF/PEEK composites under varying cooling rates remains to be elucidated. This study employed differential scanning calorimetry (DSC) combined with crystallization kinetic models including Avrami and Mo equations to analyze the non-isothermal crystallization process, while wide-angle X-ray scattering (WAXS) characterized the crystal morphology. The results indicate that with increasing PEEK melt fluidity, the crystallinity of CF/PEEK composites rose from 22.2% to 25.93% at a cooling rate of 5 °C/min, accompanied by an enhanced crystallization rate. Mechanical testing revealed that the mechanical properties improved with increasing fluidity: the tensile and flexural strengths increased from 264.8 MPa and 413.3 MPa for CF/PEEK20 to 299.1 MPa and 476.5 MPa for CF/PEEK146, respectively. Furthermore, as the PEEK melt fluidity increased, the dominant factor governing crystallization behavior shifted from chain structural stability to molecular chain mobility, and ultimately to nucleation capability.

## 1. Introduction

Carbon fiber-reinforced polyether ether ketone (CF/PEEK) composites exhibit excellent mechanical properties, thermal stability, and processability [1,2,3,4,5]. These composites are widely applied in various fields, including aerospace [6,7,8], automotive engineering [9,10,11], and robotics [12,13,14]. Mainstream processing techniques for CF/PEEK composites, such as extrusion, injection molding, and compression molding, all operate under non-isothermal thermodynamic conditions [15,16,17]. The processing protocols of CF/PEEK modulate the non-isothermal crystallization behavior, which in turn governs the ultimate product performance [18,19,20].

In previous studies, research on the crystallization behavior of CF/PEEK composites has primarily focused on the interfacial properties between CF and the PEEK matrix as well as the effects of different cooling rates. Zhong et al. [18] investigated the influence of crystalline PEEK sizing agents on the interfacial crystallization behavior and interfacial properties of CF/PEEK composites, discovering that sizing agents could induce the formation of transcrystalline structures of PEEK on carbon fiber surfaces. The CF/PEEK composite treated with only 1 wt% sizing agent exhibited an interfacial shear strength (IFSS) of 97.57 MPa, reflecting a 53.05% enhancement. Yang et al. [19] studied the effects of varying cooling rates on the interfacial crystallization behavior of T1100-grade CF/PEEK composites using POM and DSC analyses, finding that increasing the cooling rate and removing sizing agents could enhance PEEK nucleation ability and promote interfacial transcrystallization. With increasing nucleation density to approximately 0.07/μm, interfacial transcrystallinity developed in the PEEK matrix. Hachmi et al. [20] examined the crystallization kinetics of PEEK/carbon fiber composites under non-isothermal conditions and compared them with neat PEEK, employing cooling rates ranging from 0.5 to 160 °C/min. They observed that the presence of carbon fibers did not alter the fundamental crystallization mechanism but modified the crystallization temperature and rate.

However, the fluidity of materials also governs their crystallization behavior, which in turn dictates the ultimate properties [21]. Similarly, the melt fluidity of PEEK in CF/PEEK composites exerts a substantial influence on composite performance. Our previous research demonstrated that enhancing PEEK melt fluidity contributed to increased crystallinity and thereby improved composite performance [22]. Nevertheless, the underlying mechanisms governing how fluidity affects crystallization behavior and morphology in CF/PEEK remain unclear. This knowledge gap leads to an insufficient understanding of the melt fluidity–property relationship under varying processing conditions, thereby limiting the theoretical basis for optimizing product performance during processing. This study addresses this gap by establishing a quantitative fluidity–crystallinity correlation and elucidating the governing mechanisms, thereby furnishing a theoretical framework for processing optimization.

In this work, CF/PEEK composites with varying melt flow rates were prepared using PEEK resins with different melt flow rates. A full factorial experimental design was employed by controlling both the PEEK melt flow rate and cooling rate. The non-isothermal crystallization kinetics of CF/PEEK composites under different cooling rates were investigated using differential scanning calorimetry (DSC). The crystallization behavior was analyzed through the Avrami and Mo equations to elucidate the influence of PEEK melt fluidity on the crystallization process. Furthermore, the effect of PEEK melt fluidity on the final crystalline morphology of the CF/PEEK composites was examined using wide-angle X-ray scattering (WAXS). This study can provide guidance for the selection of raw materials and the design of process parameters of CF/PEEK composites by adjusting the fluidity and cooling rate of PEEK according to the different requirements of industrial production.

## 2. Results and Discussion

### 2.1. Non-Isothermal Crystallization

**Figure 1:**
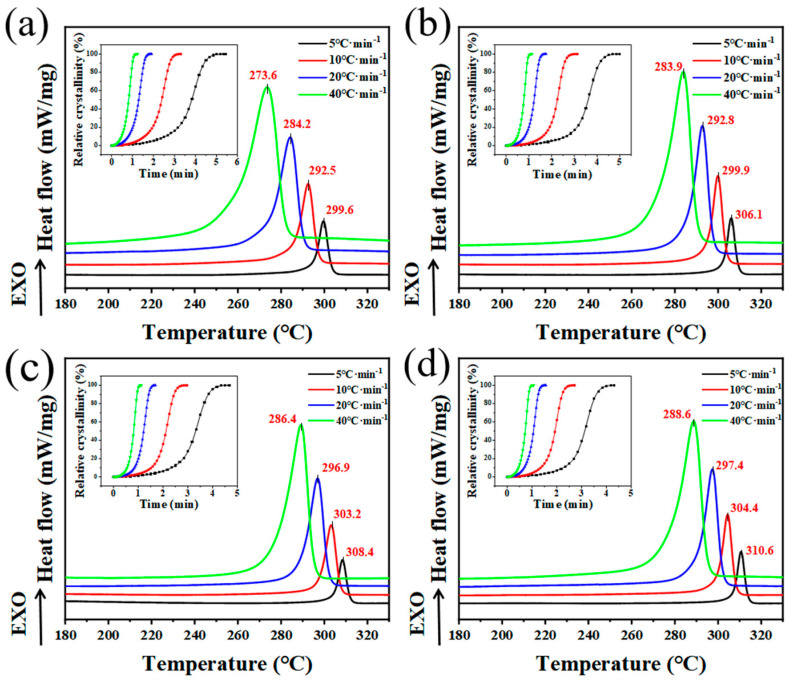
Heat flow curves and relative crystallinity versus time for different samples during crystallization: (**a**) CF/PEEK20, (**b**) CF/PEEK55, (**c**) CF/PEEK95, and (**d**) CF/PEEK146.

Non-isothermal crystallization tests were conducted at four different cooling rates. The DSC curves and relative crystallinity as a function of time are presented in Figure 1. It is evident that with the increasing melt flow rate (MFR) of PEEK, both the crystallization onset temperature and peak temperature (*Tp*) increased across all cooling rates. Specifically, *Tp* increased from 299.6 °C to 310.6 °C at the lowest cooling rate (5 °C/min), and from 273.6 °C to 288.6 °C at the highest cooling rate (40 °C/min), indicating a shift of the crystallization peak toward higher temperatures. This phenomenon is fundamentally attributed to the intrinsic differences in the raw materials; specifically, the selected PEEK with higher MFR possesses inherently lower molecular weights. This intrinsic feature enhances chain mobility and reduces the thermodynamic driving force required for crystallization, thereby enabling crystallization at smaller degrees of supercooling.

Under identical melt flow conditions, increasing the cooling rate shifted *Tp* to lower temperatures and shortened the crystallization time. Taking CF/PEEK20 as an example, as the cooling rate increased from 5 °C/min to 40 °C/min, *Tp* decreased progressively from 299.6 °C to 273.6 °C, while the crystallization time was reduced from 5.44 min to 1.23 min. The elevated cooling rate increases the degree of supercooling; however, the rate of molecular chain ordering cannot keep pace with the rapid cooling rate, preventing the polymer from achieving regular packing in a timely manner, which consequently lowers the crystallization onset temperature [23].

The relative crystallinity curves in Figure 1 all exhibited a sigmoidal (S-shaped) profile. The initial stage of crystallization is characterized by slow growth in relative crystallinity, corresponding to the nucleation-dominated period with limited crystallization rate. During the intermediate stage, crystals grow rapidly from existing nuclei, resulting in a significantly accelerated crystallization rate. In the final stage, the crystallization rate declines, likely due to the impingement and mutual compression of growing spherulites or constraints imposed by carbon fibers on crystal growth [24].

Although the maximum cooling rate in DSC analysis was limited to 40 °C/min, the fundamental kinetic trends observed here are highly translatable to industrial injection molding scenarios (>100 °C/min). Under extreme quenching conditions, the time window for crystallization is severely restricted, making the intrinsic chain mobility (dictated by MFR) the decisive factor. The enhanced crystallization capability of high-MFR PEEK identified at lab-scale cooling rates provides a robust kinetic explanation for its superior structural ordering in actual rapid manufacturing processes.

**Figure 2:**
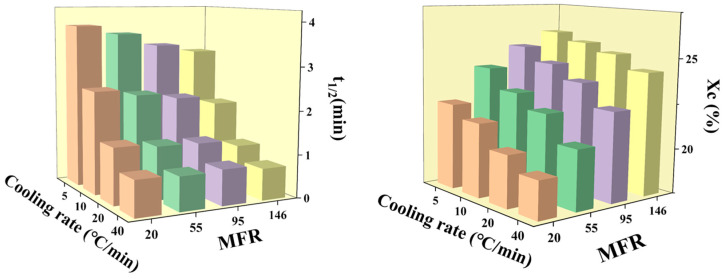
The semi-crystallization time and crystallinity of the sample at different cooling rates.

The time corresponding to 50% relative crystallinity in Figure 1 represents the half-crystallization time (*t*_1/2_) of the sample at the respective cooling rate, with the crystallinity calculated from the melting peak area. The *t*_1/2_ and degree of crystallinity (*Xc*) values are presented in Figure 2. The half-crystallization time decreased continuously with increasing melt fluidity and decreasing cooling rate. For the sample with the poorest melt fluidity (MFR 20), *t*_1/2_ ranged from 3.82 min to 0.82 min as the cooling rate increased. With improved melt fluidity (MFR 146), the maximum and minimum *t*_1/2_ values were 3.13 min and 0.72 min, respectively. At identical cooling rates, the use of PEEK with higher MFR (which corresponds to lower intrinsic molecular weights) enhanced chain mobility, enabling more rapid molecular ordering and the completion of crystallization. The influence of melt fluidity on crystallization time was particularly pronounced at lower cooling rates: at 5 °C/min, *t*_1/2_ decreased from 3.82 min for CF/PEEK20 to 3.13 min for CF/PEEK146. At high cooling rates, this difference diminished significantly because rapid cooling freezes chain segments before they can achieve regular packing.

The degree of crystallinity increased with the melt flow rate across all cooling rates. At 5 °C/min, *Xc* rose from 22.2% for CF/PEEK20 to 25.93% for CF/PEEK146, representing a relative increase of 16.8%. At 40 °C/min, *Xc* increased from 19.56% to 24.29%, corresponding to a 24.2% enhancement relative to the low-fluidity sample. Notably, the difference in crystallinity was more pronounced at higher cooling rates. Under conditions of insufficient crystallization time, high-fluidity materials with enhanced chain mobility can achieve more regular molecular packing within limited timeframes, resulting in more complete crystallization and higher ultimate crystallinity.

### 2.2. Non-Isothermal Crystallization Kinetics

#### 2.2.1. Avrami Model

The Avrami equation is commonly used to describe the isothermal crystallization kinetics of polymers [25], but it does not account for cooling rate effects in non-isothermal crystallization. Therefore, Jeziorny modified the equation to be applicable under non-isothermal conditions [26]. The crystallization process is described by the Avrami equation with parameters *n* and *Z*, as expressed by Equations (1) and (2):

(1) $$1-X_{C}(t)=\exp(-Zt^{n})$$

(2) $$\mathrm{lg}[-\ln(1-X_{C}(t))]=n\mathrm{lg}t+\mathrm{lg}Z$$

where *Xc*(*t*) is the relative crystallinity at time *t*; *Z* is the crystallization rate constant (min^−*n*^), representing the nucleation and growth rate parameters; and *n* is the Avrami exponent, which reflects the nucleation and growth mechanisms. The value of *n* equals the sum of the spatial dimension of crystal growth and the temporal dimension of the nucleation process.

To correct for cooling rate effects, Jeziorny introduced a modified rate constant, as given by Equation (3):

(3) $$\ln Ze=\frac{\ln Z}{\varphi }$$

where *Z* is the corrected crystallization rate constant for non-isothermal conditions, and *φ* is the cooling rate.

**Figure 3:**
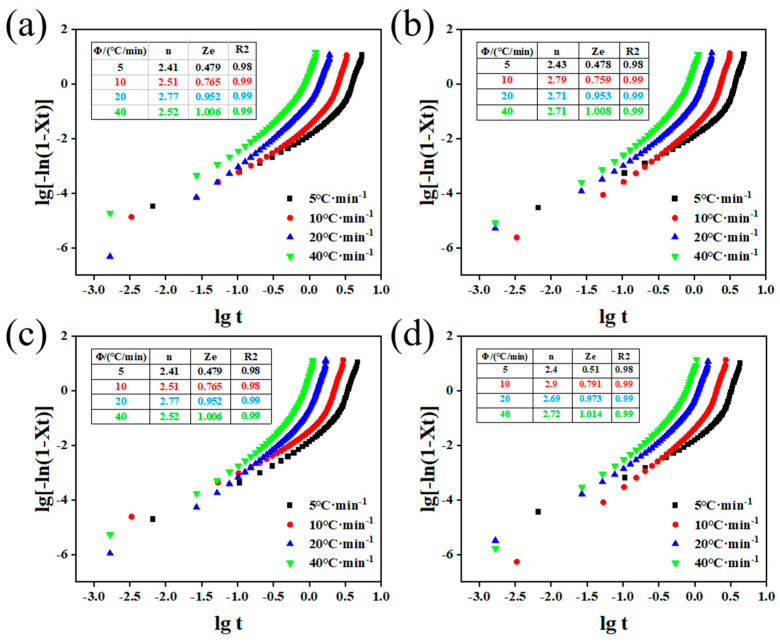
Avrami plots of lg[−ln(1 − *Xc*(*t*))] vs. lgt of the non-isothermal crystallization process of CF/PEEK composites: (**a**) CF/PEEK20, (**b**) CF/PEEK55, (**c**) CF/PEEK95, and (**d**) CF/PEEK146.

By plotting lg[−ln(1 − *Xc*(*t*))] against lgt according to Equation (2), the Avrami plots were obtained (Figure 3). Linear fitting of the curves yielded the slope and intercept, from which the Avrami exponent *n*, the crystallization rate constant *Z*, and the corrected *Ze* were calculated.

Linear fitting was strictly performed within the relative crystallinity range of 20% to 60%, avoiding the induction period at low Xt and secondary crystallization at high Xt. The correlation coefficients (R^2^) for all selected cooling rates exceeded 0.98. As shown in the tabulated data of Figure 3, the crystallization rate constant *Ze* exhibited greater variation for CF/PEEK composites with different fluidities at lower cooling rates, indicating that the crystallization rate is more sensitive to changes in melt flow rates under these conditions. Materials with a higher melt flow rate demonstrated greater crystallization rates. Conversely, at high cooling rates, *Ze* varied less significantly across different materials. This is attributed to the fact that at low cooling rates, the thermodynamic driving force provided by the environment is relatively weak, allowing materials with higher MFR and shorter molecular chains to arrange more readily into ordered crystalline structures. The Avrami exponent *n* (ranging from 2.41 to 2.79) suggests that the crystallization process follows a heterogeneous nucleation mechanism with three-dimensional spherulitic growth.

#### 2.2.2. Mo Model

The Mo equation integrates the Avrami and Ozawa equations to establish a novel model for polymer non-isothermal crystallization kinetics [27,28], formulating a quantitative relationship between the cooling rate and crystallization time at a specific degree of crystallinity. This approach overcomes the limitations of the conventional Jeziorny method and the Avrami equation, which are only applicable to the early stages of crystallization and yield rate parameters lacking clear physical significance, thereby providing a more rational theoretical framework for describing dynamic crystallization processes. The expression is as follows:

(4) nlgt+lgZ=lgK(T)−mlgΦ

**Figure 4:**
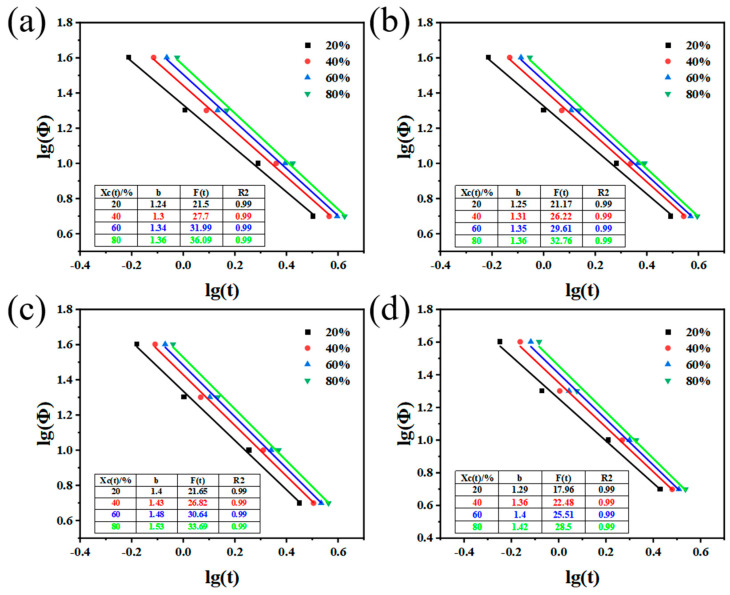
Mo plots of lgΦ vs. lgt of the non-isothermal crystallization process of CF/PEEK composites: (**a**) CF/PEEK20, (**b**) CF/PEEK55, (**c**) CF/PEEK95, and (**d**) CF/PEEK146.

It can also be expressed as:

(5) lgΦ=lgF(T)−αlgt

where *F(T)* is the cooling rate required to achieve a specific degree of crystallinity per unit crystallization time; *α* is the ratio of the Avrami exponent n to the Ozawa exponent m in non-isothermal crystallization, and *m* is the dimension of crystal growth. According to Equation (5), plotting lgΦ against lg*t* yields Figure 4.

As shown in Figure 4, data points at Xt = 20%, 40%, 60%, and 80% were used for lnΦ vs. lnt fitting. The Mo equation is relatively insensitive to secondary crystallization, and all fitted lines exhibited R^2^ > 0.99, confirming excellent linearity. A good linear relationship exists between lgΦ and lgt, indicating that the Mo equation accurately describes the non-isothermal crystallization kinetics of CF/PEEK composites. According to the physical definition of the Mo model, the parameter F(T) represents the cooling rate required to achieve a specific degree of relative crystallinity within a unit crystallization time. As shown in Figure 4, the F(T) values for CF/PEEK composites decreased systematically with increasing PEEK fluidity. For instance, to achieve 80% relative crystallinity, CF/PEEK20 requires a cooling rate of 36.09 °C/min, while CF/PEEK146 requires only 28.5 °C/min. A smaller F(T) value intrinsically signifies that the high-fluidity matrix can reach the target structural ordering with a relatively milder cooling effort, thereby confirming its faster overall crystallization rate. It is essential to distinguish this kinetic acceleration from the onset of crystallization; while the latter refers to the thermodynamic initiation (indicated by higher T_0_ and Tp in Section 2.1), the decreased F(T) validates that the entire crystallization progression was significantly accelerated by the enhanced chain mobility of the high-MFR PEEK. Both the Mo and Avrami equations revealed the same trend: the crystallization rate increased with increasing PEEK melt flow rate.

### 2.3. WAXS

**Figure 5:**
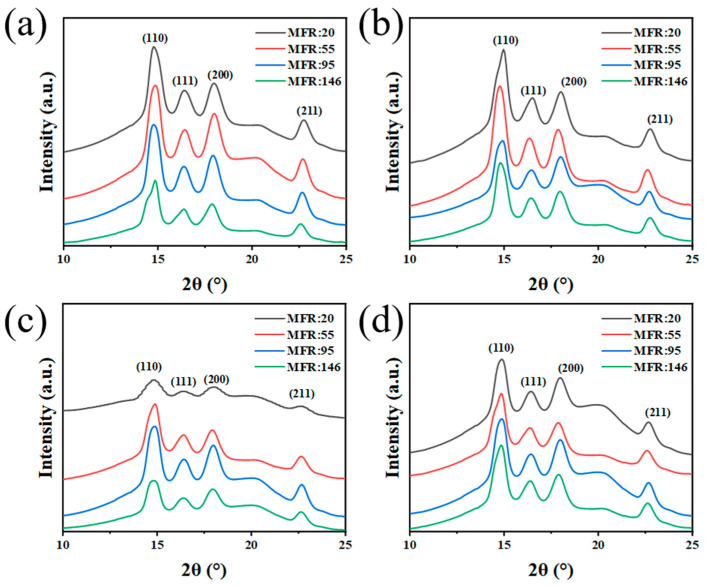
1D-WAXS curves of the CF/PEEK composites: (**a**) 5 °C/min, (**b**) 10 °C/min, (**c**) 20 °C/min, and (**d**) 40 °C/min.

**Figure 6:**
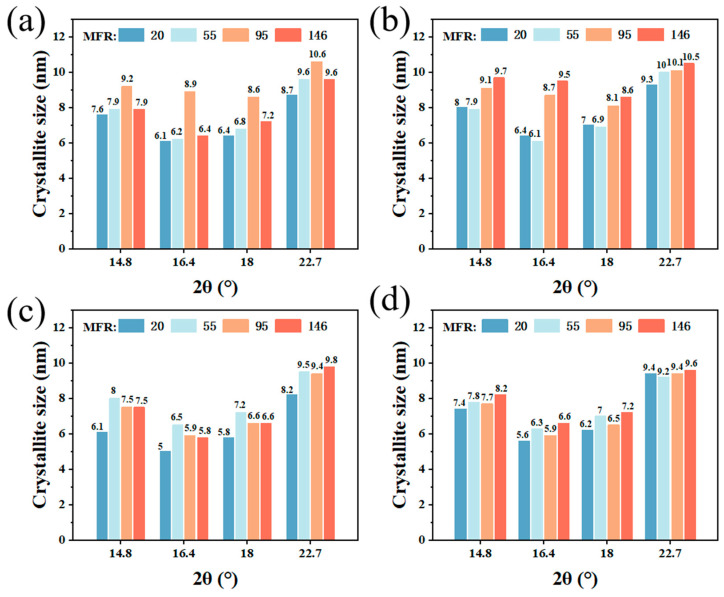
Grain size of the CF/PEEK composites at different cooling rates: (**a**) 5 °C/min, (**b**) 10 °C/min, (**c**) 20 °C/min, and (**d**) 40 °C/min.

WAXS was employed to characterize the variations in the crystal morphology of CF/PEEK composites at different cooling rates, with the 2D-WAXS patterns of the composites shown in Figure S1. The 2D-WAXS images were reduced to 1D-WAXS scattering profiles via azimuthal integration using Fit2D V17.006 software, as shown in Figure 5. The resulting patterns revealed that all samples retained the characteristic α-crystalline phase, exhibiting distinct diffraction peaks at 2*θ* = 14.8°, 16.4°, 17.9°, and 22.7°, which corresponded to the (110), (111), (200), and (211) crystallographic planes, respectively. This indicates that variations in melt flow rate and cooling rate did not affect the crystal form of PEEK. It should be noted that the measurements were conducted using an incident X-ray wavelength of 1.24 Å; consequently, the diffraction angles exhibited a systematic shift toward lower values compared to those typically obtained with standard Cu Kα radiation (λ = 1.5418 Å), consistent with Bragg’s law (*nλ* = 2*d*sin*θ*). The crystallite sizes (Figure 6) were subsequently determined through peak profile fitting of the diffractograms using PeakFit V4.12 software.

At the lowest cooling rate (5 °C/min), the crystallite size exhibited a non-monotonic trend, initially increasing and then decreasing. Taking the (110) plane with the strongest diffraction intensity as an example, the crystallite sizes were 7.6 nm, 7.9 nm, 9.2 nm, and 7.9 nm, respectively, reaching a maximum at MFR 95. Upon further increase in PEEK melt flow rate, the crystallite size decreased. At low cooling rates, the melt resides in the high-temperature growth region for a sufficient duration, providing adequate time for molecular chain disentanglement, diffusion, and nucleation-growth processes. As MFR increased from 20 to 95, the degree of molecular chain entanglement decreased and melt fluidity improved, facilitating the ordered arrangement of molecular chains and enabling the full growth of crystallites. When MFR further increased to 146, the excessively short molecular chains led to an excessively high nucleation density. Consequently, crystals impinged upon each other without sufficient space for growth, resulting in reduced crystallite size.

As the cooling rate increases, the growth time available for crystallites decreases, diminishing the controlling effect of chain structural stability on the crystallization process. Consequently, melt fluidity becomes the dominant factor governing crystallization. For low-fluidity materials, the highly entangled molecular chains cannot rapidly disentangle and diffuse to active sites to complete the filling of crystal nuclei, severely restricting crystallite growth and resulting in smaller crystallite dimensions. In contrast, high-fluidity materials possess shorter molecular chains with reduced entanglement density, enabling higher efficiency in chain segment diffusion and reorganization. This facilitates more rapid growth within limited crystallization timeframes, leading to continuously increasing crystallite sizes.

At the highest cooling rate (40 °C/min), the excessively fast cooling rate caused substantial overlap between nucleation and growth processes, minimizing the regulatory effect of molecular chain fluidity. Higher MFR materials possess smaller segmental steric hindrance, enabling faster crystallization even within extremely short timeframes. The crystallite size thus exhibited a slow and modest increasing trend, with (110) plane crystallite sizes of 7.4 nm, 7.8 nm, 7.7 nm, and 8.2 nm, respectively. Should the cooling rate continue to increase, the crystallization time would be further compressed; the difference in crystallite sizes would diminish while maintaining a slight increasing trend.

**Figure 7:**
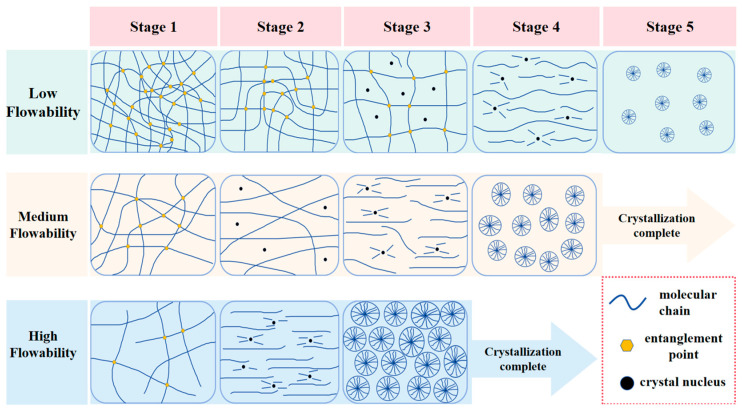
Schematic illustration of the mechanism by which PEEK with different melt flow rates affects the crystallization behavior of CF/PEEK composites.

The effect of PEEK melt flow rate on the crystallization behavior of CF/PEEK composites is shown in Figure 7. In the first stage of the crystallization process, PEEK molecular chains with different flowabilities exhibit distinct morphologies. Low-fluidity PEEK chains are highly entangled with numerous entanglement points, resulting in extremely low chain mobility. Medium-fluidity PEEK chains are only partially entangled and thus exhibit moderately restricted mobility. In contrast, high-fluidity PEEK chains contain fewer entanglements and possess high segmental mobility. As crystallization proceeds, the chains in high-fluidity PEEK undergo nucleation and rapidly grow into spherulites, whereas the chains in medium- and low-fluidity PEEK primarily undergo disentanglement and begin to align into preliminarily ordered structures. In the third stage, spherulites in the high-fluidity PEEK matrix grow rapidly and become more perfected; medium-fluidity PEEK completes the initial nucleation and enters the crystal growth stage; whereas low-fluidity PEEK has only just formed ordered chain arrangements and is preparing for nucleation. Ultimately, in the fourth and fifth stages, medium- and low-fluidity PEEK complete the crystallization process successively.

Due to its strong segmental mobility, high-fluidity PEEK exhibits rapid crystal growth following nucleation, ultimately achieving high crystallinity with larger crystal dimensions within a shorter timeframe. Conversely, low-fluidity PEEK suffers from difficult disentanglement and slow crystal growth, becoming completely frozen before full development, resulting in lower final crystallinity and smaller crystal dimensions.

### 2.4. Mechanical Properties

**Figure 8:**
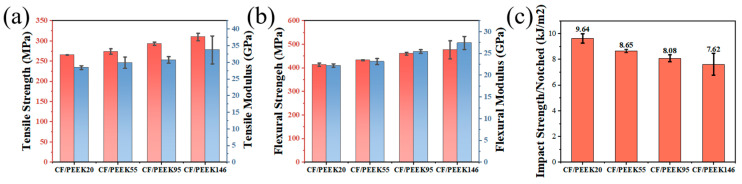
Mechanical properties of the CF/PEEK composites with different flowabilities: (**a**) tensile, (**b**) flexural, and (**c**) impact properties.

To validate the applicability of the kinetic analysis in practical industrial scenarios, injection molding experiments were conducted, characterized by ultra-high cooling rates (>100 °C/min). The test results are presented in Figure 8.

To verify the practical significance of the non-isothermal crystallization kinetic analysis in actual processing, the CF/PEEK composite specimens were prepared via injection molding (cooling rate > 100 °C/min). Their mechanical properties are presented in Figure 8. Experimental results indicate that the tensile and flexural strengths of the CF/PEEK composites increased significantly with the increasing melt flow rate (MFR) of the matrix. Specifically, the tensile and flexural strengths of CF/PEEK20 were 264.8 MPa and 413.3 MPa, respectively. In contrast, the high-fluidity CF/PEEK146 exhibited the most outstanding mechanical performance, achieving a tensile strength of 299.1 MPa (tensile modulus of 33.7 GPa) and a flexural strength of 476.5 MPa (flexural modulus of 27.4 GPa). This performance enhancement is primarily attributed to the synergistic effect of optimized crystallization behavior and advantageous processing dynamics. As the core focus of this study, the variation in crystallinity directly dictates the intrinsic strength of the matrix. High-fluidity PEEK, characterized by shorter molecular chains and lower entanglement density, exhibits significantly enhanced chain mobility. Under extremely high cooling rates during processing, this elevated mobility allows the molecular chains to arrange into the crystal lattice more rapidly within a restricted timeframe, thereby achieving a higher degree of crystallinity (Xc increasing from 22.2% to 25.93%) and a more perfected crystal morphology. Tests on the pure PEEK matrices further confirmed that within the investigated MFR range, the strengthening effect induced by the increased crystallinity overwhelmingly surpassed the negative impact of molecular weight reduction, enabling the matrix to provide superior mechanical support. Building upon the matrix crystallization strengthening, improved processing dynamics provide additional guarantees for composite performance. During the high-shear melt-compounding process, the highly fluid melt exhibits lower internal friction, effectively reducing the shear stress exerted on the continuous carbon fibers [29]. This low-viscosity environment mitigates mechanical damage and attrition of the fibers, thereby preserving a longer residual fiber length in the final composites [30]. Concurrently, the low-viscosity melt possesses superior impregnation dynamics, significantly enhancing the interfacial adhesion between the matrix and the CFs. The combination of a more perfected crystalline structure, effective fiber length retention, and more robust interfacial adhesion collectively constitutes the exceptional mechanical load-bearing capacity of CF/PEEK146.

In contrast to the enhanced static load-bearing capabilities (tensile and flexural strengths), the impact strength of the composites exhibited a distinct downward trend with increasing matrix fluidity (Figure 8c). The notched impact strength decreased from 9.64 kJ/m^2^ for CF/PEEK20 to 7.62 kJ/m^2^ for CF/PEEK146. This phenomenon is governed by a complex interplay of matrix ductility, microstructural evolution, and interfacial mechanics. Fundamentally, the increase in MFR corresponds to a reduction in the intrinsic molecular weight of the PEEK matrix. This reduction leads to a lower concentration of molecular chain entanglements, which drastically diminishes the intrinsic ductility and plastic energy-dissipation capability of the polymer upon sudden impact. Furthermore, the elevated degree of crystallinity and the perfected crystal structure further restrict the localized yielding and deformation of the matrix, exacerbating its embrittlement. While the high-fluidity matrix successfully preserves residual fiber length and enhances fiber–matrix interfacial adhesion—which is highly beneficial for steady tensile loading—these rigid constraints further impede the plastic deformation of the already brittle matrix under high-speed impact events, facilitating rapid crack propagation. Therefore, the low-fluidity (MFR 20) system, benefiting from higher molecular weight and extensive chain entanglements, retains superior toughness and impact resistance, providing a crucial design alternative for applications requiring high damage tolerance.

Based on the aforementioned mechanisms and the performance parameters of CF/PEEK, this study can provide valuable references for the production of certain industrial products. Taking the specimens in this study as examples, MFR95 combined with 5 °C/min represents the optimal balance between crystallite size and crystallinity. This parameter combination yielded the largest crystallite size accompanied by relatively high crystallinity, resulting in excellent crystal structural integrity, which suits applications with specific demands for crystal structural regularity. The MFR146 + 5 °C/min combination, featuring the highest crystallinity across all groups together with smaller crystallite dimensions, emerged as the optimal choice for mechanical properties, delivering superior tensile and flexural strength and modulus, applicable to high-end civil structural components. At the high cooling rate of 40 °C/min, the crystallite sizes remained virtually identical across all melt fluidity grades; notably, MFR146 + 40 °C/min maintained relatively high crystallinity, striking a balance between molding efficiency and fundamental mechanical properties, suitable for large-scale high-efficiency industrial production. Although the low-fluidity MFR20 system exhibited inferior crystallinity and crystallite size compared with medium- and high-fluidity systems at all cooling rates, and its poor melt fluidity poses processing challenges, its corresponding higher molecular weight and extensive molecular chain entanglement confer potential advantages in impact toughness and fatigue resistance. This system can serve as a specialty-grade raw material for applications with modest strength requirements but specific toughness demands, compatible with molding processes such as compression molding and autoclave curing that impose less stringent requirements on melt fluidity, thereby complementing medium- and high-fluidity systems.

## 3. Materials and Methods

### 3.1. Materials

Continuous carbon fiber tows (TZ800S, T800 grade-12K) were obtained from China Weihai Tuozhan Fiber Co., Ltd. (Weihai, China). The average fiber diameter was approximately 5.5 μm (Figure S2). PEEK resins with four different MFR (20, 55, 95, 146 g/10 min) (Figure S3) were purchased from Hairuite Engineering Plastics Co., Ltd. (Jiamusi, China).

### 3.2. Fabrication of CF/PEEK Composites

PEEK pellets were dried in an air-circulating oven (Yiheng, Shanghai, China) at 150 °C for 3 h. Subsequently, the PEEK matrix and the continuous CF tows were melt-compounded at a mass ratio of 7:3 in a twin-screw extruder. The continuous CF tows were fed directly into the extruder melt zone, where they were subjected to severe shear forces and chopped into short fibers during the blending process. The extruder rotational speed was set to 80 rpm, followed by pelletization to obtain composite pellets. The composite materials were named CF/PEEK20, CF/PEEK55, CF/PEEK95, and CF/PEEK146 based on their distinct PEEK melt flow rates. The dispersion state of CF within the CF/PEEK composites is illustrated in Figure S4, demonstrating uniform distribution in the PEEK matrix without evident agglomeration.

### 3.3. Characterization

#### 3.3.1. Thermal Property Testing of CF/PEEK Material

**Figure 9:**
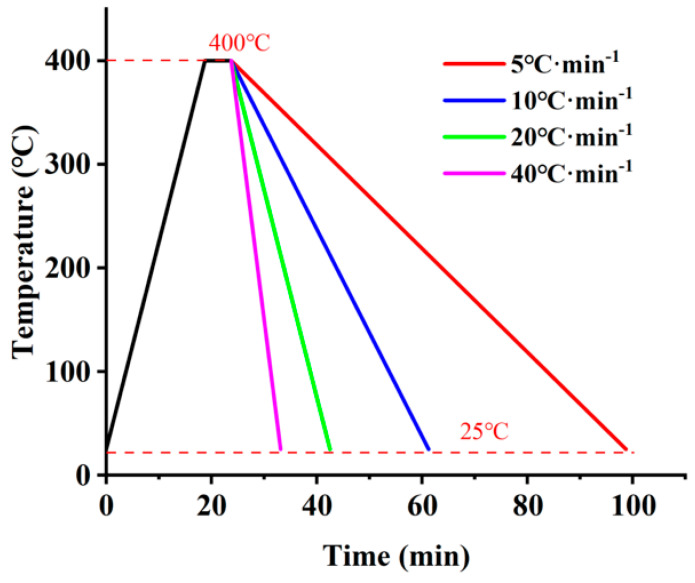
Schematic of the heating and cooling process for sample preparation.

The crystallization kinetics were investigated using a differential scanning calorimeter (DSC, DSC 300 Caliris) (NETZSCH, Shanghai, Germany). All experiments were performed under a nitrogen flow rate of 40 mL/min. First, the samples were heated to 400 °C at a rate of 20 °C/min and held at 400 °C for 5 min to eliminate thermal history. Subsequently, the samples were cooled down to 25 °C at different cooling rates of 5, 10, 20, and 40 °C/min, respectively, followed by reheating to 400 °C at a rate of 20 °C/min for a second melting process. The heat flow curves were recorded and analyzed. The schematic diagram of the first melting-crystallization process is shown in Figure 9.

The crystallinity of the CF/PEEK composites was calculated by the DSC results using Formula (6):

(6) $$Xc=\frac{\Delta Hm-\Delta Hc}{\Delta H_{0}}\times \frac{100\%}{\omega_{PEEK}}$$

where *X*c represents the degree of crystallinity of the CF/PEEK composite; Δ*H*m is the melting enthalpy; Δ*H*c is the cold crystallization enthalpy; Δ*H*_0_ is the theoretical melting enthalpy of 100% crystalline PEEK, typically 130 J/g for PEEK [31]; and ω_PEEK_ denotes the mass fraction of PEEK in the composite.

#### 3.3.2. WAXS

The 2D-WAXS data were collected at the BL16B1 beamline of the Shanghai Synchrotron Radiation Facility (SSRF) using an incident wavelength of 1.24 Å, using a Pilatus 2M detector (DECTRIS, Baden, Switzerland). The sample-to-detector distance was set to 200 mm. Each sample was exposed for 200 s. The obtained WAXS data were analyzed using Fit2D and PeakFit V4.12 software. Based on the Scherrer Equation (7), the crystallite size (*D_hkl_*) of the CF/PEEK materials was calculated.

(7) $$D_{hkl}=\frac{K\lambda }{\left(\beta_{hkl}\times \cos\theta_{hkl}\right)}$$

where *K* is the Scherrer constant (0.89); λ is the X-ray wavelength (1.24 Å); *β_hkl_* is the full width at half maximum (FWHM) of the diffraction peak.

#### 3.3.3. Mechanical Property Testing

Mechanical test specimens were prepared by injection molding. Tensile and flexural properties were measured at room temperature on an Instron 5966 universal testing machine (Norwood, MA, USA) according to the ISO 527 [32] and ISO 178 [33] standards, respectively. Notched Charpy impact strength was tested at room temperature using a HIT-2492 impact testing machine (Jinjian Detection Equipment Co., Ltd., Chengde, China) in accordance with ISO 179 [34]. For each mechanical test (tensile, flexural, and impact), at least five independent specimens were tested for each composition. The results are reported as the average values accompanied by their standard deviations.

## 4. Conclusions

This study investigated the effects of PEEK melt fluidity on crystallization behavior, crystal morphology, and material properties of CF/PEEK composites. As the melt fluidity of PEEK in CF/PEEK composites increased, the crystallization process shifted toward higher temperatures across all cooling rates. At 5 °C/min, the crystallization peak temperature rose from 299.6 °C to 310.6 °C, the degree of crystallinity increased from 22.2% to 25.93%, and the crystallization rate was enhanced, enabling the material to achieve the same relative crystallinity at lower cooling rates. The mechanical property test results exhibited the same trend as the crystallinity changes: with increasing fluidity, the tensile and flexural strengths improved from 264.8 MPa and 413.3 MPa for CF/PEEK20 to 299.1 MPa and 476.5 MPa for CF/PEEK146, respectively.

It was found that at lower cooling rates, the crystallite size of CF/PEEK composites first increases and then decreases with increasing melt fluidity. As the cooling rate increases, this trend gradually transitions to monotonic increase, and finally to slow increase with small variation at high cooling rates. This indicates that the dominant factor governing crystallization behavior shifts from chain structural stability prevailing over fluidity, then to fluidity prevailing over structural stability. Ultimately, at high cooling rates, the restricted chain mobility severely hinders polymer diffusion; consequently, the instantaneous nucleation rate becomes the absolute dominant factor, superseding the synergistic mechanisms of both nucleation and diffusion-driven crystal growth. By investigating how PEEK melt fluidity affects the crystallization mechanisms, we can provide more detailed guidance for CF/PEEK processing techniques, thereby achieving better performance control.

## Data Availability

The original contributions presented in this study are included in the article. Further inquiries can be directed to the corresponding authors.

## Supplementary Materials

The following supporting information can be downloaded at: https://www.mdpi.com/article/10.3390/molecules31111810/s1, Figure S1: 2D-WAXS patterns of CF/PEEK composites prepared under varying MFR and cooling rates; Figure S2: The surface morphology of T800 CF; Figure S3: The melt flow rate (MFR) of the PEEK and CF/PEEK composite; Figure S4: SEM micrographs of the fracture surfaces from tensile test specimens; Figure S5: The second heating curves of CF/PEEK composites (a) CF/PEEK20, (b) CF/PEEK55, (c) CF/PEEK95, (d) CF/PEEK146; Figure S6: Heat flow curvesfor different samples during crystallization (a) CF/PEEK55, (b) CF/PEEK95.
