# Supplementary material for: Effect of Polyether Ether Ketone Melt Fluidity on Crystallization Behavior of Carbon Fiber Reinforced Polyether Ether Ketone Composites
Source: Molecules. 2026 May 25;31(11):1810. doi: 10.3390/molecules31111810 (PMC13257583; doi:10.3390/molecules31111810)
Supplement: Supplementary file 1 [file molecules-31-01810-s001.zip › molecules-4256744-supplementary.pdf]

Effect of PEEK Melt Fluidity on Crystallization Behavior of CF/PEEK Composites

Weifeng Liu [1], Xiaran Miao*[2], Shiwen Tao [1], Ji Li [1], Jianzhong Ma [1,3], Jinjun Yang [3]，Hui Li *[1]

1 State Key Laboratory for Modification of Chemical Fibers and Polymer Materials, Center for Advanced Low-dimension Materials, College of Materials Science and Engineering, Donghua University, Shanghai 201620, China

2 Shanghai Synchrotron Radiation Facility, Shanghai Advanced Research Institute, Chinese Academy of Sciences, Shanghai 201204, China

3 Avic Composite Co., Ltd, Beijing 101300, China

Correspondence: miaoxr@sari.ac.cn(XR.M.); lihui@dhu.edu.cn (H.L.);

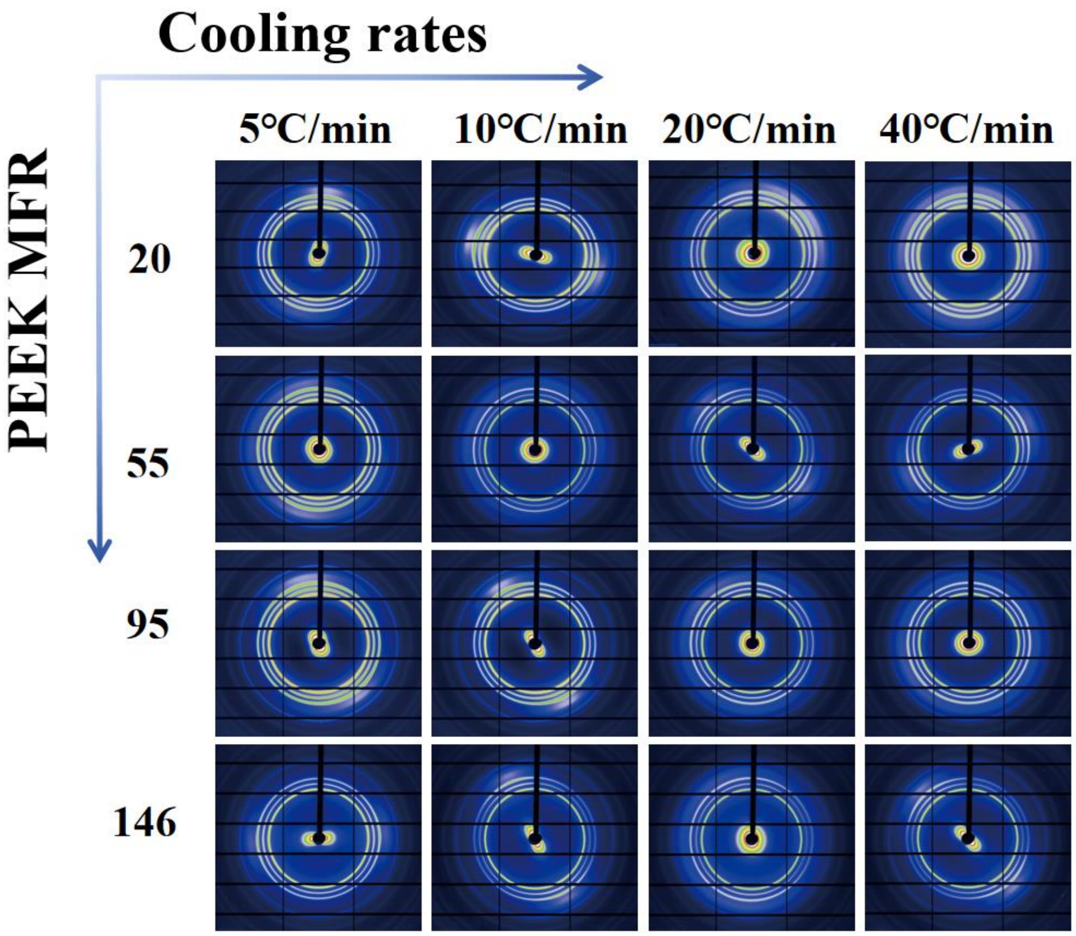

Figure S1. 2D-WAXS patterns of CF/PEEK composites prepared under varying MFR and cooling rates are presented in Figure. S4. Well-defined diffraction rings corresponding to distinct crystallographic planes are observed across all samples, indicating the formation of well-developed crystalline structures in the PEEK matrix. The invariant positions of these diffraction rings further confirm the preservation of PEEK's original crystal form, i.e., no polymorphic transformation occurred under the investigated processing conditions.

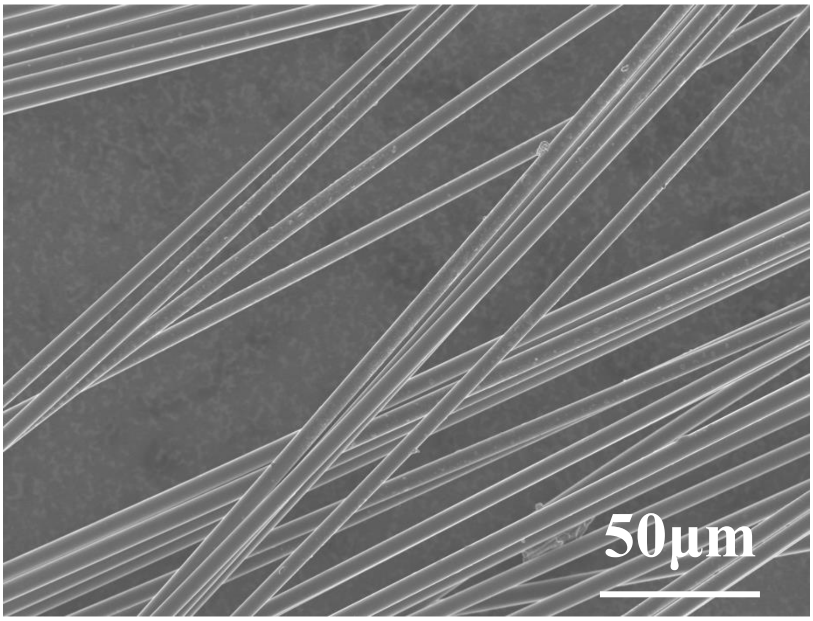

Figure S2. The surface morphology of T800 CF was characterized by the scanning electron microscopy (SEM, Regulus8230, Hitachi, Japan) operating under an acceleration voltage of 5 kV. exhibiting a smooth surface devoid of grooves, characteristic of dry-process carbon fibers, with a fiber diameter of approximately 5 μm.

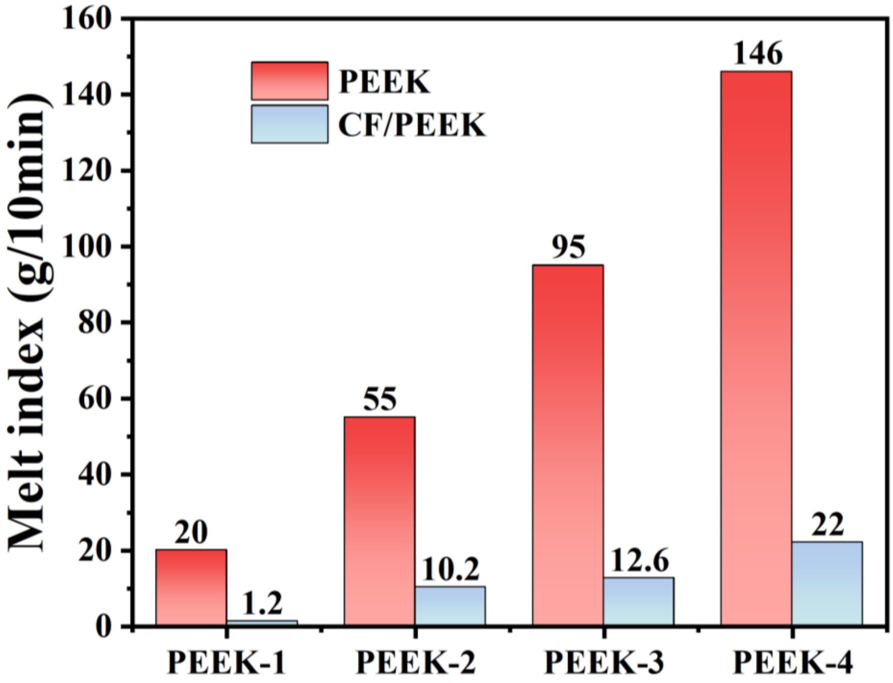

Figure S3. The melt flow rate (MFR) of the PEEK was measured using a melt flow rate tester (Model FSB-400, Xiamen Fubusi Testing Equipment Co., Ltd.). The melt flow rates of the four PEEK materials, ranked from low to high, were 20, 55, 95, and 146 g/10 min, respectively. The melt flow rates of the four CF/PEEK composite

materials, ranked from low to high, were 1.2, 10.2, 12.6, and 22 g/10 min, respectively.The measurements were conducted under the conditions of 380 °C, a load of 5 kg, and a holding time of 5 minutes.

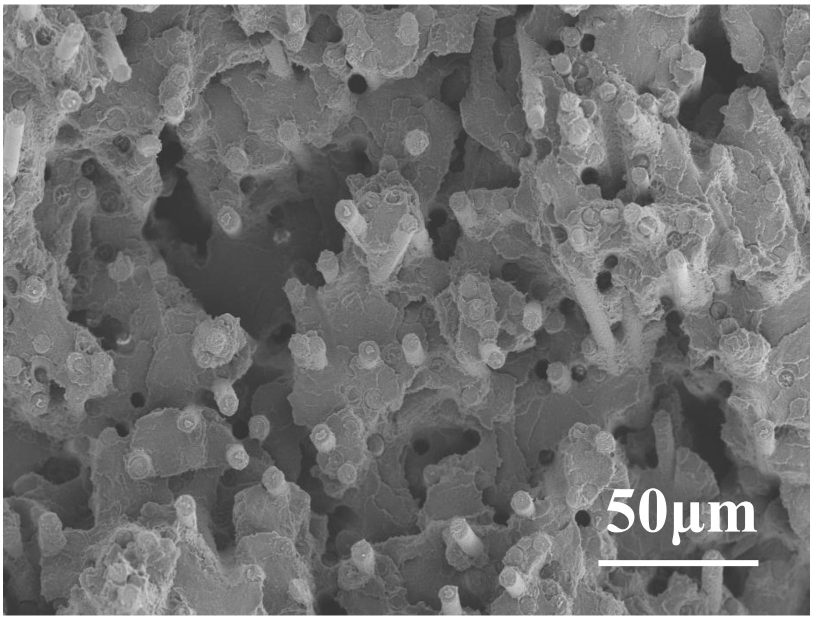

Figure S4. SEM micrographs of the fracture surfaces from tensile test specimens are shown in Figure. S3. The images reveal a uniform distribution of carbon fibers within the PEEK matrix, with no evident fiber agglomeration or void formation observed in the prepared CF/PEEK composites.

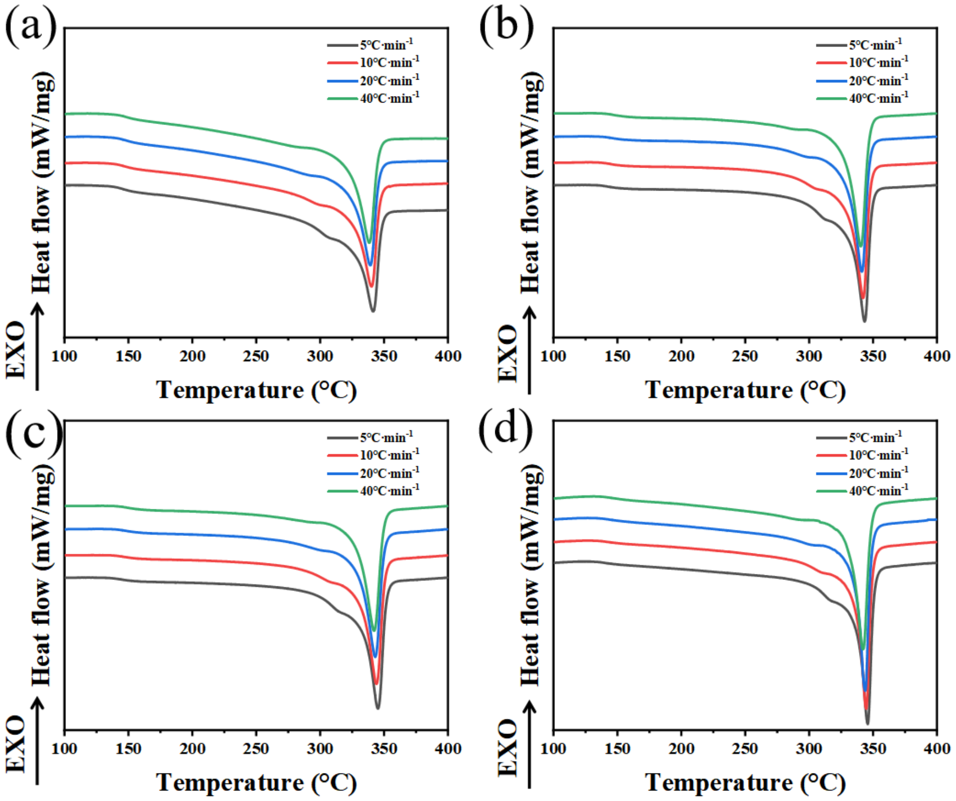

Figure S5 the second heating curves of CF/PEEK composites (a) CF/PEEK20, (b) CF/PEEK55, (c) CF/PEEK95, (d) CF/PEEK146. For (high-fluidity samples, no discernible cold crystallization peak is observed, indicating that the enhanced molecular chain mobility enables the PEEK matrix to fully crystallize during the cooling phase. Conversely, for low-fluidity samples, a slight cold crystallization peak becomes noticeable just prior to the melting endotherm, suggesting that the restricted chain mobility limits the extent of crystallization during cooling, leaving residual amorphous regions that subsequently recrystallize upon reheating.R[2]

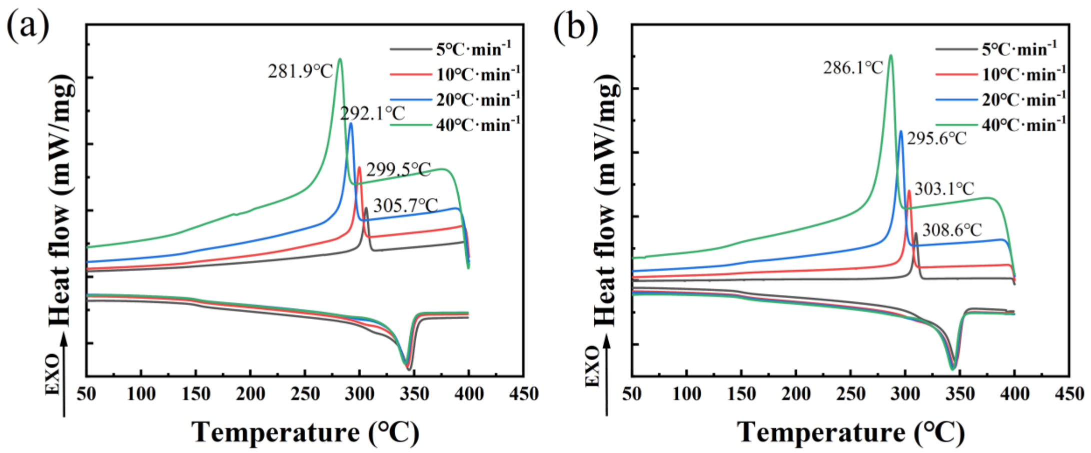

Figure S6 Heat flow curvesfor different samples during crystallization (a)

CF/PEEK55, (b) CF/PEEK95. The comparative analysis clearly reveals that the addition of CF significantly elevates both the onset crystallization temperature (T0) and the peak crystallization temperature (Tp) compared to neat PEEK under identical cooling conditions. These observations are fully consistent with our preliminary data, which already demonstrated that CF incorporation increases the degree of crystallinity. Together, these features provide robust evidence that carbon fibers act as highly efficient nucleating agents via heterogeneous nucleation, accelerating the overall crystallization process, even though the rigid fibers might partially restrict the long-range mobility of PEEK molecular chains.
